# Are clinicians being prepared to care for abused women? A survey of health professional education in Ontario, Canada

**DOI:** 10.1186/1472-6920-9-34

**Published:** 2009-06-18

**Authors:** C Nadine Wathen, Masako Tanaka, Cristina Catallo, Adrianne C Lebner, M Kinneret Friedman, Mark D Hanson, Clare Freeman, Susan M Jack, Ellen Jamieson, Harriet L MacMillan

**Affiliations:** 1 Faculty of Information & Media Studies, The University of Western Ontario, London, Ontario, Canada; 2 Department of Psychiatry and Behavioural Neurosciences, McMaster University, Hamilton, Ontario, Canada; 3 School of Nursing, McMaster University, Hamilton, Ontario, Canada; 4 Faculty of Health Sciences, McMaster University, Hamilton, Ontario, Canada; 5 Michael G. DeGroote School of Medicine, McMaster University, Hamilton, Ontario, Canada; 6 Interval House of Hamilton-Wentworth, Hamilton, Ontario, Canada; 7 Departments of Pediatrics, McMaster University, Hamilton, Ontario, Canada

## Abstract

**Background:**

The current project undertook a province-wide survey and environmental scan of educational opportunities available to future health care providers on the topic of intimate partner violence (IPV) against women.

**Methods:**

A team of experts identified university and college programs in Ontario, Canada as potential providers of IPV education to students in health care professions at the undergraduate and post-graduate levels. A telephone survey with contacts representing these programs was conducted between October 2005 and March 2006. The survey asked whether IPV-specific education was provided to learners, and if so, how and by whom.

**Results:**

In total, 222 eligible programs in dentistry, medicine, nursing and other allied health professions were surveyed, and 95% (212/222) of programs responded. Of these, 57% reported offering some form of IPV-specific education, with undergraduate nursing (83%) and allied health (82%) programs having the highest rates. Fewer than half of undergraduate medical (43%) and dentistry (46%) programs offered IPV content. Postgraduate programs ranged from no IPV content provision (dentistry) to 41% offering content (nursing).

**Conclusion:**

Significant variability exists across program areas regarding the methods for IPV education, its delivery and evaluation. The results of this project highlight that expectations for an active and consistent response by health care professionals to women experiencing the effects of violence may not match the realities of professional preparation.

## Background

Almost one in ten women in North America are physically abused by an intimate partner in any given year, and up to a third of women in population-based surveys report some form of physical or emotional abuse over the course of their lifetime [1-3]. Women exposed to intimate partner violence (IPV) suffer significant acute and chronic physical and mental health outcomes, including death, injury, chronic pain, poor gynaecologic and general health outcomes [4-6], posttraumatic stress disorder, depression, anxiety and substance abuse [5,7-9]. Women who experience violence use more health care services [10-12] yet are at risk for marginalization in the health care system due to the complexity of their physical and mental health needs [13].

In the face of this often overwhelming problem, the health sector has been identified as having a key role in identifying and responding to abused women [14,15], and, despite ongoing debate regarding the evidence for effective clinical responses [16-18], health care providers (HCPs) across the spectrum of disciplines are increasingly encouraged to ask about exposure to violence as part of their routine care of women, especially when clinical indicators of abuse are present [9,19].

The health sector, however, has been criticized for providing less than optimal care to women exposed to IPV. As Alpert [20] states " [t]he ability of most health professionals to effectively identify, assess, and respond to domestic violence has lagged far behind societal awareness and community responses" (p. 666). This is in part due to HCP perceptions that abuse is rare in their practice or that IPV identification and referral are not part of their role or responsibilities [21], as well as specific barriers to addressing IPV, including: lack of time to deal with a complex social issue; a fear of offending patients or retribution from the abuser; personal experiences with family violence; feeling helpless when clients disclose/experience violence; and not knowing how to recognize, ask about and respond to IPV [20-27]. These barriers, many of which can be attributed to lack of education and training, affect the HCP's confidence in her/his ability to provide appropriate responses to women experiencing violence [21]. Though the relative lack of well-conducted evaluative research assessing the impact of IPV education on clinical knowledge, skills and practices remains a pressing gap [27], current research does suggest an association between IPV training and clinical behaviour [28]. A survey of over 900 nurses and physicians in Ontario, Canada found that formal training in IPV was significantly related to whether they routinely initiated discussions about IPV with patients, and yet almost 60% in both professions reported never having received such education [29].

Trends in IPV education have been described based on surveys of U.S. and Canadian medical and nursing schools. In a 1989 survey of 116 US and Canadian medical schools regarding training about all types of adult domestic violence [27], the Centers for Disease Control and Prevention (CDC) found that 53% of respondents did not offer any training while 42% offered training as part of at least one required course. Approximately ten years later, Alpert et al. [30] surveyed medical school deans and students from across the US and found that at that time, 60% of respondents reported course offerings on IPV. However, while increases in the number of schools providing some IPV education is a positive step, these surveys do not provide in-depth information on the type and extent of content provided to learners. In a survey of Canadian nursing schools (n = 155), the topic of IPV did not receive as many scheduled hours of instruction in the curriculum as child abuse and suicide [31], which was consistent with findings in a US study [32]. While there are emerging trends in the use of more integrative alternatives to primarily lecture-based pedagogy [33-36], such as problem-based learning, the usual approach to IPV education, certainly in medical schools, has been stand-alone lectures not truly integrated into important clinical training experiences [30]. For example, in the Canadian survey by Ross et al. [31] there was little indication of planned clinical experiences related to IPV.

Evaluative data regarding IPV education in allied health professional programs is lacking, and a quasi-systematic review by the Institute of Medicine's Committee on Training Needs of Health Professionals to Respond to Family Violence [37] concludes that across health professions " [i]n formal curricula on family violence, content is incomplete, instruction time is generally minimal, content and teaching methods vary, and the issue is not well integrated throughout the educational experience" (p. 44).

The surveys described above demonstrate that while there is some increase in IPV education in undergraduate health care professional programs, barriers persist to the implementation and evaluation of such training. These barriers include lack of institutional endorsement, lack of funding to support new curriculum, and competition for curricular time between and within departments [38].

Advocates of a more consistent and comprehensive response by the health sector to abused women have argued that all HCPs interacting with women should be prepared to recognize, inquire about and respond to cases of abuse [37]. To better understand whether and how HCPs are prepared in this area, the current project surveyed HCP undergraduate and post-graduate educational programs in Ontario, Canada.

## Methods

### Setting and Definitions

Ontario is a Canadian province of over 12.5 million people with a publicly funded health care system that allocates about CDN$35 billion per year for health services, including over 150 hospitals, 22,000 practicing physicians, 140,000 nurses, and over 40,000 practitioners registered in the allied health professions we surveyed. It has 18 publicly funded universities, 6 with medical schools (5 at the time of this study) and 24 colleges of applied arts and technology.^i ^For clarity, and consistent with the way these programs are generally considered in Canadian universities, primary degrees obtained in medicine (MD), nursing (BScN) and allied professions such as occupational and physical therapy (BSc(OT) and BSc(PT)) are considered as "undergraduate" while training beyond the primary professional designation (e.g., MScN, residency programs, etc.) are considered "post-graduate" education. College-level degrees and diplomas were also considered "undergraduate" education.

### Sampling and Procedures

All potential university and college programs in relevant areas (Table 1) were identified and a database of potential respondents created. A number of programs within a university or college were often grouped under one of our "program types" (e.g., undergraduate allied health programs included midwifery, BScN and occupational therapy, undergraduate medical programs included the general MD program, including both pre-clerkship and clerkship curricula). Results are presented using the following groups: allied health, dentistry, medicine and nursing.

**Table 1:** Description of Surveyed Program Types

**Program Type**	**Level**	**Description**
Allied Health	UG	University midwifery baccalaureate programs, social work baccalaureate and physical and occupational therapy baccalaureate programs. For colleges this includes social service worker programs, child and youth worker programs, aboriginal social service programs, police service worker programs.
Allied Health	PG	Masters/PhD programs for social work, psychology, physical and occupational therapy
Dentistry	UG	Doctor of dental surgery (DDS) degree and college-based dental hygiene programs.
Dentistry	PG	Specialty/residency areas for dentistry such as oral and maxillofacial surgery, prosthodontics, public health, and forensic dentistry.
Medicine	UG	All medical doctor (MD) degree programs.
Medicine	PG	Specialty/residency areas for medicine including: family medicine, psychiatry, pediatrics, emergency medicine, surgery, obstetrics and gynecology, internal medicine and community health. MD/PhD programs for these medical specialties were also included.
Nursing	UG	For the general classification of Registered Nurse (RN), this includes baccalaureate programs, combined/collaborative college/university baccalaureate programs, and post-RN to baccalaureate programs. For training of Registered Practical Nurses, college programs for practical nursing were included.
Nursing	PG	For extended classification (Nurse Practitioner), this includes all primary and acute Nurse Practitioner programs, Masters and PhD programs in nursing and health sciences and all continuing education and certification offered at colleges or universities.

UG = undergraduate; PG = postgraduate

For each program, individuals with sufficient knowledge of the curriculum to complete the survey were identified as eligible respondents. In October 2005, all respondents were mailed a survey with an invitation to participate in a brief telephone interview. Trained interviewers then contacted respondents by telephone, inviting them to schedule an interview time. The telephone survey involved use of a standard, scripted version of the survey. If the participant did not respond to initial follow up, a multi-modal follow up method based on Dillman [39] was implemented using mail, email and telephone.

On occasion, multiple respondents were identified. For example, when considering a specific program, there may have been several individuals who could have been interviewed, including the Dean, the Assistant Dean, the Director and the Program Coordinator. All of these individuals were sent a survey and contacted for follow-up, but it was generally only the Program Coordinator, knowing enough detail regarding elements of the curriculum, who was designated by the program to complete the survey. The unit of analysis for response rate calculations and descriptive statistics, therefore, was the individual program, not the number of surveys sent.

When a respondent was not aware of IPV-related educational initiatives in their program, they were asked to recommend another individual who could answer on the program's behalf. On many occasions, respondents provided a number of contacts, all of whom were followed up by interview staff. In cases where more than one person contributed to completion of the survey for a specific program, this was still counted as one completed survey. When inconsistencies arose among respondents, the interviewer conducted further interviewing until consensus was achieved.

Interviews were conducted from October 2005 to March 2006. The majority were completed by telephone and lasted from 15 to 45 minutes. Five respondents completed their survey by mail; they were then contacted by phone by an interviewer to review their responses for any additional information. Programs were also asked to send copies of, or otherwise provide access to, any written course descriptions or formal policies or other curricular documents specific to IPV education. Ethical approval for this study was deemed unnecessary by the Research Ethics Board of McMaster University.

### Survey Instrument

The survey instrument [see Additional file 1] was developed by the research team, with input from IPV and methods experts, to assess whether and how IPV education was offered. More specifically, the survey first inquired whether IPV-related education was offered and if so, how this was done (e.g., as a required course, clinical practicum, etc.), by whom, and with what resources. Additional questions asked about: specific IPV content; acknowledgement for completing training; integrating IPV content into the overall curriculum; facilitators and barriers to IPV content delivery; and existing evaluation strategies to assess the quality of this education. The questions were generally closed-ended, with the list of answers read to the respondent; however most questions included an "other" category where respondents could provide answers outside the scope of those listed; the interviewer also asked an open-ended question soliciting additional comments on each question and noted responses in a space provided on the form. To ensure completeness and usability and determine approximate administration time, the survey was pilot-tested with a group of university/college-based program administrators in local programs that did not meet inclusion criteria for the study (i.e., were not in the specified health and allied health education areas). Adjustments to the instrument and/or the telephone survey process were made accordingly.

### Data Analysis

Survey responses were entered into a Microsoft Access database, which was then imported into SPSS Version 12.0 for generation of the descriptive statistics reported here. All data were entered by one research assistant and verified by two others prior to analysis. Any uncertainties in data entry were resolved through consensus after reviewing the initial data collection forms and, if necessary, discussing the data with the interviewer who conducted that interview. As the goal of this project was to describe the provision of IPV education to future HCPs in Ontario, the focus was on providing descriptive summaries of the data.

## Results

### Provision of IPV Education

Table 2 shows the response rates for each program type. Overall, 95% of eligible programs responded to the survey (range 88% to 100%). Table 2 also summarizes responses, by program type, to the primary question regarding provision of IPV education, with 57% (n = 120) of the responding sample indicating that at least some IPV education was being offered at the time of the survey. The findings described below relate to specific aspects of IPV education based on information from programs that responded "yes" to the IPV provision question, and included programs from all types except postgraduate dentistry.

**Table 2:** Response Rate and Provision of IPV Education by Program Type

**Program Type**	**Level**	**Eligible programs sent survey N**	**Programs responding to survey % (n)**	**Programs providing IPV education % (n)**
Allied Health	UG	66	98% (65)	82% (53)
Allied Health	PG	34	94% (32)	28% (9)
Dentistry	UG	13	100% (13)	46% (6)
Dentistry	PG	2	100% (2)	0% (0)
Medicine	UG	8	88% (7)	43% (3)
Medicine	PG	39	92% (36)	25% (9)
Nursing	UG	41	98% (40)	83% (33)
Nursing	PG	19	89% (17)	41% (7)
**Total**	**UG**	**128**	**98% (125)**	**76% (95)**
**Total**	**PG**	**94**	**93% (87)**	**28.7% (25)**
**Grand Total**		**222**	**95% (212)**	**57% (120)**

UG = undergraduate; PG = postgraduate

Across program types the following content areas were generally covered by over 85% of responding programs: overview of IPV, risks for IPV, characteristics of victims and perpetrators, methods for identification of IPV, interventions, and availability of and access to community resources. At the level of individual programs, these content types were generally provided by over 80% of programs, with the only outlier being the undergraduate dentistry programs. A third or fewer of responding programs indicated that they covered the topics related to risks, characteristics, identification or intervention; however all of these programs provided an overview and discussed community resources.

Regarding time spent on IPV content, respondents indicated that an average of one to four hours was spent covering each of the content categories described above, as well as models, frameworks and theories that examine IPV, gender, multicultural and indigenous groups, and, to a lesser extent (i.e., averaging less than one hour), issues specific to violence by women against men, violence in same sex relationships, and common couple violence.

We also assessed how IPV content is provided, that is, whether it is included in required or elective courses (or other approaches) and what teaching techniques are used to deliver the content. Table 3 shows that IPV content is rarely delivered within required or elective courses or practica dedicated solely to IPV. Rather, it was often a component of a required or elective course, or delivered by other means, primarily through workshops, and, to a lesser extent, through distance education, online materials, or video-conferencing. In terms of credit for this content, 69% (81/117, 3 no response) of all program areas reported providing at least some form of credit or acknowledgement to learners upon completion of IPV-related education, with the majority of programs offering partial credit (i.e., credit given for a course in which IPV content was a component). Postgraduate nursing (43%, or 3/7) and undergraduate allied health (36% or 18/51, 2 no response) programs were notable in that over a third of these types of programs offered a full course credit specific to IPV content. Over 30% (35/117, 3 no response) of respondents across all program areas reported that no course credit or acknowledgement was offered.

**Table 3:** IPV Education Delivery Methods

**Program Type**	**Level**	**Required course % (n)**	**Required practicum % (n)**	**Required part of a course % (n)**	**Elective course % (n)**	**Other means of delivery % (n)**
Allied Health	UG	34% (17)*	12% (6)*	64% (32)*	24% (12)*	62%(31)*
Allied Health	PG	0% (0)^†^	0% (0)**	57% (4)^†^	43% (3)**	29% (2)^†^
Dentistry	UG	0% (0)	0% (0)	100% (6)	0% (0)	50% (3)
Medicine	UG	0% (0)	0% (0)	66% (2)	0% (0)	67% (2)
Medicine	PG	22% (2)	11% (1)	22% (2)	22% (2)	89% (8)
Nursing	UG	9% (3)^‡^	6% (2)^‡^	66% (21)^‡^	3% (1)^‡^	56% (18)^‡^
Nursing	PG	14% (1)	0% (0)	43% (3)	14% (1)	29% (2)
**Total (all program types)**		**20% (23)**	**8% (9)**	**61% (70)**	**17% (19)**	**58% (66)**

UG = undergraduate; PG = postgraduate; *3 no response; ^† ^2 no response; ^‡^1 no response

Table 4 presents the teaching methods used by those who responded to this question (approximately one-third of programs did not respond to this item). In this case, standardized patients referred to actors paid to perform the role of a patient so that students may practice clinical skills. These are differentiated from simulated learning experiences, which included case study, role play and interviewing. IPV-related resources included audio visual (AV) materials, texts and/or learning kits.

**Table 4:** Teaching Methods for IPV Content by Program Type

**Program Type**	**Level**	**Problem-based IPV Scenario % (n) (# no response)**	**Standardized Patient IPV Scenario % (n) (# no response)**	**Other simulated learning % (n) (# no response)**	**Use of IPV-specific materials % (n) (# no response)**
Allied Health	UG	63% (27) (10)	28% (11) (13)	68% (28/41) (12)	79% (33) (11)
Allied Health	PG	20% (1) (4)	17% (1) (3)	50% (3) (3)	50% (3) (3)
Dentistry	UG	25% (1) (2)	75% (3) (2)	50% (2) (2)	100% (4) (2)
Medicine	UG	50% (1) (1)	50% (1) (1)	50% (1) (1)	100% (2) (1)
Medicine	PG	40% (2) (4)	20% (1) (4)	33% (2) (3)	17% (1) (3)
Nursing	UG	42% (8) (14)	14% (3) (12)	47% (9) (14)	90% (17) (14)
Nursing	PG	25% (1) (3)	25% (1) (3)	75% (3) (3)	80% (4) (2)
**Total (all program types)**		**50% (41) (38)**	**26% (21) (38)**	**59% (48) (38)**	**76% (64) (36)**

UG = undergraduate; PG = postgraduate

### Resources for Delivering IPV Content

We asked respondents to indicate what type of faculty (full-time, part-time, sessional or external experts) delivered IPV content to learners. Key findings include that most programs (84%), with the exception of postgraduate nursing (43%), have primarily full-time faculty teaching IPV content. Overall, 38% of programs used part-time faculty (range 32% to 56%). The greatest proportion of sessional or contract faculty utilized for this content was found in the allied health undergraduate (41%) and post-graduate (56%) programs. All programs reported some use of experts in the area to deliver content (range 17% to 57%, mean 49%).

Table 5 summarizes some of the key resources we asked about regarding delivery of IPV content. In addition to these resources; 67.5% of respondents indicated that written policies, procedures, curricula or syllabi regarding IPV-related education existed; 79% reported some form of extra-organizational collaboration (i.e., IPV or non-IPV related), and 62% reported collaboration regarding IPV education specifically.

**Table 5:** Instructional Tools and Resources for Delivering IPV Content

**Program Type**	**Level**	**Training on faculty/department goals** **% (n)**	**Support to attend IPV conferences** **% (n)**	**Opportunities for collaboration** **% (n)**	**Budget for IPV materials** **% (n)**
Allied Health	UG*	41% (20)	78% (38)	86% (42)	65% (32)
Allied Health	PG	0% (0)	22% (2)	22% (2)	11% (1)
Dentistry	UG	50% (3)	66% (4)	83% (5)	50% (3)
Medicine	UG	0% (0)	33% (1)	66% (2)	66% (2)
Medicine	PG**	50% (4)	25% (2)	88% (7)	25% (2)
Nursing	UG*	21% (6)	59% (17)	72% (21)	38% (11)
Nursing	PG	29% (2)	14% (1)	43% (3)	29% (2)
**Total (all program types) (9 no response)**		**32% (35)**	**59% (65)**	**74% (82)**	**48% (53)**

UG = undergraduate; PG = postgraduate; *(4 no response); **1 no response

### Factors Influencing Delivery of IPV Education

We asked respondents about 'internal' influences within their faculty or department and 'external' influences outside their faculty or department that impacted the provision of IPV-related education. Results across program areas are presented in Table 6. Of note, an issue across program types, both internally and externally, was availability of funding for IPV education. Also, time available within the curriculum was likely to be identified as an internal barrier to delivery of IPV education across programs and in particular across graduate-level programs.

**Table 6:** Impact of Internal and External Influences on IPV Education Provision Across All Programs

**Internal Influences**	**Mean** **(sd)**	**'Facilitator' % (n)**	**'Neutral' % (n)**	**'Barrier' % (n)**
a. Availability and access to instructors who teach IPV content	1.65 (0.97)	81.0% (85)	12.4% (13)	6.7% (7)
b. Adequate instructor preparation for teaching IPV content	1.96(1.16)	72.4% (76)	13.3% (14)	14.3% (15)
c. The instructor's commitment to IPV content	1.41(0.73)	91.3% (94)	5.8% (6)	2.9% (3)
d. Access to instructors with specific IPV-related expertise/research endeavours	2.03(1.17)	65.1% (69)	24.5% (26)	10.4% (11)
e. Faculty/department commitment to IPV education	1.91(0.99)	69.8% (74)	24.5% (26)	5.7% (6)
f. Valid and relevant faculty/department mission or goals related to IPV	2.44(1.36)	53.5% (53)	23.2% (23)	23.2% (23)
g. Opportunities for curriculum renewal/revision	1.63(0.96)	82.9% (87)	11.4% (12)	5.7% (6)
h. Culture of the faculty/department	1.71(0.85)	78.4% (80)	19.6% (20)	2.0% (2)
i. Receptiveness of education recipients to IPV content	1.58(0.78)	88.6% (93)	9.5% (10)	1.9% (2)
j. Funding allocation for IPV education	3.07(1.46)	37.0% (37)	21.0% (21)	42.0% (42)
k. Access to resources to deliver IPV related content (e.g. A/V equipment)	1.73(1.10)	80.0% (84)	10.5% (11)	9.5% (10)
l. Adequate amount of time to include IPV content in curriculum (length of program)	2.78(1.39)	44.8% (47)	19.0% (20)	36.2% (38)
**External Influences**				
a. Current political climate in your institution	1.83 (0.95)	77.4% (82)	15.1% (16)	7.5% (8)
b. Current political climate more broadly	2.12 (1.12)	64.2% (68)	23.6% (25)	12.3% (13)
c. Health care restructuring	2.66 (1.27)	39.8% (39)	33.7% (33)	26.5% (26)
d. Accreditation	2.08 (1.08)	63.9% (62)	26.8% (26)	9.3% (9)
e. Licensing body approval process	2.11 (1.16)	58.7% (54)	30.4% (28)	10.9% (10)
f. External funding	2.83 (1.43)	38.5% (37)	21.9% (21)	39.6% (38)

Responses on a 5-point scale: '1' = facilitator to '5' = barrier; facilitator = responses of '1' or '2', neutral = responses of '3', barrier = responses of '4' or '5'

### Evaluation

Respondents were asked whether and how they evaluated learners' acquisition of the IPV content. Overall, 59% (71/120, 38 missing) reported that they assessed what students learned from the IPV curriculum. The most common approach (72% of programs) used a test or examination that including IPV-related questions. Other approaches included learner self-evaluation (28%), a general test or exam (not IPV-specific) (45%); and verbal reports by learners (51%). Respondents were also asked whether there were any procedures in place to evaluate the quality of IPV education provided in their program overall. Approximately half (51/103, 17 no response) reported that there were.

### Summary of Collected Curricula

Curricula received from respondents were analyzed using a template designed for this purpose (available from the authors). Across all program areas, only 10 course curriculum descriptions (8 from college programs), one training manual and various PowerPoint presentations were available for review. Many programs at the university level stated that they did not posses formal, written curricula for the IPV content provided within a part of a course, therefore the curricula reviewed tended to outline separate courses that were specific to violence.

In general, review of this sample confirmed the survey results regarding the scope of IPV content and the methods for content delivery (lectures, simulated patients, guest experts, etc.). Some courses included all forms of family violence (i.e., child abuse, elder abuse and IPV), while others focused solely on IPV. Some courses included special topics (like violence among individuals with disabilities, violence within the workplace, the role of the media, women's health and social policy). A few courses included examination – from a number of theoretical perspectives – of broader socio-political structures that influence both the perpetration of violence and policy responses to it.

Courses varied in how they evaluated student learning; two included a general test or examination with the inclusion of IPV questions, three had an essay with IPV available as an optional topic and one course required an IPV-specific essay. Other forms of evaluation not identified as IPV-specific were: student and group presentations, online assignments, resource development, journal or reflective exercises, media clip assessment and volunteer work.

## Discussion

In Ontario, significant variability exists across program areas regarding the methods for IPV education, its delivery and evaluation. While over 80% of undergraduate allied health and nursing programs provide at least some IPV-related education, fewer than half of undergraduate medicine and dentistry programs reported providing this kind of education, while the proportions for postgraduate programs were generally lower. This suggests that physicians, one of the key types of HCPs encouraged to intervene in cases of IPV [14], may not have sufficient educational opportunities in their primary training to become adequately knowledgeable, clinically competent, or comfortable in assessing and responding to IPV [27,29,37]. The same can be said for other allied health care professionals represented in this sample.

In terms of specific IPV content, all programs providing IPV education reported covering a general overview of the issue, its risk factors, characteristics of victims and perpetrators and information regarding local community resources; this finding is consistent with the literature in this area [27,40]. The average teaching time spent on these topics across all program areas ranged from one to four hours per year. Some program areas also reported teaching about identifying and responding to IPV. Given the generally limited curricular content (usually < 4 hours), and the fact that many programs reported no formal evaluation (nor in most cases credit or acknowledgement) of what students learned in this area, little is known regarding the uptake of this knowledge and whether or not it influences clinical practice. This lack of rigorous evaluation has been cited as an important gap in knowledge [27,37], with the result that evidence-based clinical core competencies for caring for abused women remain unknown [37].

Significant variability existed in the methods for IPV education delivery in this sample. Across programs, the most common approach to including IPV content in the curriculum was as a required component of a course, or delivery of content through regular or occasional workshops, and less commonly, using distance or online techniques. In terms of what this means for future HCPs, specific cases can be used to illustrate key points. For example, the small proportion of postgraduate medical programs with required IPV education means that only 11% of (4 of 36 responding) programs required medical postgraduate trainees to receive any IPV education. On the other hand, 63% of allied health undergraduate programs had required IPV education. While resourcing and other barriers may explain why some areas lag well behind others in formalizing their offerings in this area, findings such as these highlight the fact that providing IPV content is still generally ad-hoc and informal. Although integrated, longitudinal, multi-disciplinary and experiential approaches to IPV education as advocated by experts in this area [20,27,30,38] may be emerging in some programs, they are not yet evident, with one exception^ii^, in Ontario's post-secondary institutions.

The ability to provide content consistently in any area will depend on the availability of expert, or at least knowledgeable, faculty. Program areas reported differing degrees of resource support – including availability of full- versus part-time faculty – for IPV education. Allied health and nursing programs generally reported more material support, including budget to acquire IPV-specific educational materials and access to necessary AV equipment. While our cross-sectional data do not allow inferences regarding whether access to appropriate faculty and resources impacts how IPV education is delivered, this is an important area for further study.

Many program areas, especially medicine and nursing, stated that there are multiple competing core educational interests that may make IPV content less of a priority than other areas. When asked what kinds of factors facilitate IPV education delivery, access to instructors with IPV expertise, opportunities for curriculum renewal and content receptiveness within the department and among learners were key influences. Respondents identified lack of funding and lack of adequate time for IPV content in the curriculum as important barriers. External influences on IPV education included the current political climate within the institution, and more broadly, health care restructuring and accreditation, as well as licensing approval processes.

Results from our review of available curricular documents may shed some light on how 'formalized' IPV content is within these programs. The fact that we received few curricular documents may suggest that IPV content is viewed as a component of more widely ranging courses. This may indicate that it is offered in a less formal and structured way, as also reflected in the common statement from respondents that IPV content is present but not in the form of written, formalized syllabi. However the fact that we received so few documents makes it difficult to provide firm conclusions regarding this issue. These results are consistent with surveys of nursing [31] and medical [30] programs. According to the survey by Alpert and colleagues [30], 86% of medical school deans reported inclusion of IPV-related content in their curricula, but only 55% of their medical students reported awareness of this topic as part of their courses. The extent to which lack of a more formal curricular status for IPV content may be related to fewer resources and, perhaps, less student focus, warrants further consideration.

### Limitations & Future Research

This survey was conducted in one jurisdiction within a publicly-funded health care system, and with the majority of responding programs being from publicly-funded or -assisted post-secondary institutions. However our excellent response rate, and the fact that the sampling frame was in fact the entire population of relevant programs, leads us to believe that the results provide a reasonable description of the state of IPV education for students training to be health care providers in Ontario, and that this result is, at least to some extent, generalizable to other Canadian provinces, and perhaps more broadly.

We intentionally included programs that educate students training to be the types of HCPs who are expected – at least in the discourse of the field – to identify and respond to abuse among their female patients or clients. In making these decisions we may have cast the net too widely – in some cases, respondents who indicated "no" to provision of any IPV content also expressed confusion as to why we would be contacting them in the first place. It may be that some such programs felt incorrectly identified – so much so that they did not feel it even appropriate to respond. However, given our selection criteria, this is an interesting observation in its own right: there may be assumptions made among those advocating for how abused women should be cared for in the health sector that are at odds with the actual practices in this sector. This observation requires further exploration to ensure a better match between what advocates and women expect, and what HCPs and the health system are able to provide.

The cross-sectional nature of our research, and its specific focus, leave unresolved many key questions that require further consideration and evaluation. These include: 1) How much IPV education are students provided relative to other content areas in their primary educational programs? The present survey gives us an idea of what is offered with respect to IPV education, but how this compares to other content areas within each of these programs is not known. 2) What is the relative quality of IPV education provided among the different program areas, within and between program types, and does this lead to any differences in HCP skills and practices the long run? 3) What, specifically, are the core knowledge and skills required by HCPs in the identification, assessment and response to women exposed to violence? 4) What specific training exists at the local level – i.e., within institutions or when professionals receive orientation to new hospitals, clinics or other settings? 5) To what extent do educational administrators (e.g., deans and other leaders within educational institutions) recognize the importance of IPV-related education in curricula? More broadly, the question remains as to whether and how IPV education delivered in primary educational programs actually improves clinicians' assessment of and response to abused women.

## Conclusion

The present study highlights the gap between expectations often cited in the IPV literature regarding an active and consistent response to abused women from the health care sector, and the realities of health professionals' educational experiences and preparation. If clinicians are expected to appropriately identify and respond to abused women, they must be provided with relevant education. While this is becoming increasingly available in some areas of primary professional education, it is not yet available to all those in training.

Current literature and expert opinion suggest that health professional training regarding IPV must be multi-faceted, examine key individual and social risk factors correlated with violence and provide insight into special considerations for women of diverse backgrounds and geography [40,41]. Programs that can be tailored to the practice context may be most likely to succeed [42]. Brief educational interventions, such as short workshops, have generally, with some exceptions [28], not been shown effective in improving clinicians' recognition of or response to abused patients, though overall knowledge immediately following these interventions does increase [43,44]. Our findings, consistent with those of others [20,27,37] indicate that an integrated approach is rarely the case among the more than 200 programs we surveyed in Ontario. Multifaceted training should provide clinicians with the tools to help women and men understand and where possible avoid the risks for IPV, identify IPV when clinical signs and symptoms are present, and intervene (either directly or through community referrals) with women experiencing it, addressing the acute and chronic physical and mental health effects of violence.

IPV education must also consider the context of clinical work settings and address common barriers to IPV assessment and response. A number of educational resources specific to IPV in health care now exist [45], with an emerging emphasis on evaluating programs and curricula as they are implemented. For example, Moscovic et al. [46] randomized medical students to receive either didactic training alone or didactic training plus experiential community-based training among medical students and found that those with the added "real-world" experience felt much better prepared to provide clinical care in this area. The use of new media to improve access for students and practitioners also shows promise [47,48]. Finally, some countries have implemented national level training and evaluation to ensure that clinicians in both training and practice have access to education in this area [49].

In short, there is hope; if we consider the field of child abuse education, which was similarly marginalized in health professional education just a few decades ago [50,51] and is now a formal component of most programs [30], then, as Hamberger [27] (p. 223) states "*the future shows promise that the next generation of physicians [and other health care providers] will understand and accept IPV and its health effects on their patients as constituting an important health issue that they will comfortably address*."

## Competing interests

The authors declare that they have no competing interests.

## Authors' contributions

CNW, HLM and CF conceived of the study and obtained funding. CNW and MT drafted the manuscript. CC oversaw data collection and entry. MT oversaw data cleaning and preparation, assisted by MKF and AL. EJ participated in the design of the study and performed the statistical analysis. MDH and SMJ participated in study design and data interpretation and helped to draft the manuscript. All authors read and approved the final manuscript. McMaster IPV Education Research Team (below) provided guidance regarding the objectives and design of the research.

## Pre-publication history

The pre-publication history for this paper can be accessed here:



## Supplementary Material

Additional file 1 **Survey Instrument**. The survey instrument used to collect data from the university and college programs participating in the study. Click here for file
