# Supplementary material for: Are clinicians being prepared to care for abused women? A survey of health professional education in Ontario, Canada
Source: BMC Med Educ. 2009 Jun 18;9:34. doi: 10.1186/1472-6920-9-34 (PMC2709616; doi:10.1186/1472-6920-9-34)
Supplement: Additional file 1 — Survey Instrument. The survey instrument used to collect data from the university and college programs participating in the study. [file 1472-6920-9-34-S1.pdf]

INTIMATE PARTNER VIOLENCE EDUCATION SURVEY

Below is the definition of Intimate Partner Violence (IPV) that the team is using for this project:

The Centers for Disease Control defines intimate partner violence against women (IPV) as any behaviour purposely inflicted by a male or female partner against a woman within an intimate relationship that causes physical, psychological or sexual harm. Such behaviour includes acts of physical aggression, psychological or emotional abuse, as well as forced intercourse and other forms of sexual coercion. According to the World Health Organization, violence is multifaceted and includes individual, relationship, community and societal determinants. Current evidence suggests that there are differences between men and women who experience IPV in terms of both risks for, and consequences of, violence. While the prevalence and consequences of IPV tend to have greater impact on the health and well-being of women, violence against men may also occur within intimate relationships. Therefore, we have included questions concerning education that may cover this type of violence as well.

Description of Education Provided

1. Does your faculty/department offer any education to learners regarding intimate partner violence (IPV)?

☐ Yes *– Please proceed to question 2.*
☐ Unsure *– Our interviewer will ask for a contact that we can follow up with who will be able to answer this question*
☐ No *– Finished, thank you.*

2. a) Does your faculty/department have written policies and/or procedures regarding IPV education?

☐ Yes
☐ No

b) Does your faculty/department have curriculum descriptions and/or course syllabi for the IPV education?

☐ Yes
☐ No

c) Would your faculty/department be willing to share these policies and procedures, curricula and/or syllabi with our research team?

☐ Yes
☐ No

For those respondents who say they have and are willing to share written policies and procedures:

What's the best way to send us a copy?

☐ Email electronic copies to doddp@mcmaster.ca
☐ Available on a website (get URL: ____)
☐ Fax to 905-383-8068
☐ Send by our courier company
☐ Have us send you a stamped, self-addressed envelope for you to mail them to us
☐ Other? ____

3. How is the IPV education delivered? ***Please consider all years of your program – we want to know about all types of delivery, so you can check off more than one option.***

- ☐ Required course
- ☐ Required practicum
- ☐ Required segment of a course
- ☐ Elective course
- ☐ Core theme throughout a required course
- ☐ Core theme throughout an elective course
- ☐ Video-conferencing or video-streaming materials
- ☐ Other: ____
- ☐ Regularly offered workshop
- ☐ Regularly offered distance education
- ☐ Occasional distance education
- ☐ Web-based or on-line materials
- ☐ Not covered
- ☐ Future plans to add

4. a) What, if any, of these topics or approaches are included in the IPV education?

Check all that apply related to IPV	**YES**	**NO**	**PLAN TO INTRODUCE**
a. General overview of IPV against women	☐	☐	☐
b. Specific details about IPV risk factors or correlates	☐	☐	☐
c. Specific details about characteristics of IPV victims and perpetrators	☐	☐	☐
d. Specific knowledge to aid learners in identifying IPV	☐	☐	☐
e. Specific knowledge to aid learners in responding to IPV (e.g. interventions)	☐	☐	☐
f. Present information about local community resources	☐	☐	☐
g. Discuss issues specific to gender	☐	☐	☐
h. Discuss issues specific to multicultural communities (e.g. cultural inclusion, language barriers, immigration)	☐	☐	☐
i. Discuss issues specific to indigenous communities	☐	☐	☐
j. Utilize a model, framework or theory to guide education on IPV (e.g., gender analysis, feminist theory)<br>If yes, specify: _____________________________	☐	☐	☐
k. Discuss IPV by women against men	☐	☐	☐
l. Discuss IPV in same sex relationships	☐	☐	☐
m. Discuss common couple violence (e.g. violence by both partners against one another)	☐	☐	☐

5. **Is formal credit or acknowledgement provided to those who complete the IPV education? (check all that apply)** *We would like an overview of the range of credit options available to learners. Any other options that are not available here can be added.*

- ☐ Yes, as follows:
  - ☐ Full course credit
  - ☐ Partial course credit
  - ☐ CME credit
  - ☐ Certificate of completion
  - ☐ Acknowledgment for accreditation purposes
  - ☐ Other, specify: ______________________________
- ☐ No
- ☐ Other, specify: ______________________________

6. **Who provides the IPV education? [by professional, we mean faculty / staff with credentials in a health or social service field, such as medicine, nursing, social work, etc.)** *Again, a general overview is all that we are interested in for this question. Select all that apply.*

- ☐ Full-time professional faculty
- ☐ Part-time professional faculty
- ☐ Sessional or contract professionals
- ☐ Staff from other faculties/areas of the university/college
- ☐ Experts in the area of IPV from the community
- ☐ Full-time non-professional staff
- ☐ Part-time, sessional or contract employees
- ☐ Other, specify: ____________________________

7. **What resources do those delivering the IPV education receive from your faculty/department?**

- ☐ Specific training on overall educational goals of faculty/department
- ☐ Specific training on the topics covered in the IPV education
- ☐ Support to attend professional conferences related to IPV
- ☐ Opportunities for those delivering education to collaborate or share ideas
- ☐ Allocated budget to purchase teaching aids related to IPV (e.g. videos, simulated patients))
- ☐ Audio visual equipment (e.g. LCD projectors, TV/VCRs)
- ☐ None (No resources related to IPV are provided)
- ☐ Future plans to add resources related to IPV education
- ☐ Other, specify: ____

Evaluation of IPV Education

8. **Are there any procedures in place to evaluate the quality of IPV education in your faculty/department?**

- ☐ Yes
- ☐ No
- ☐ Other, specify: ____________________________

9. **Please rate the following "<u>internal influences</u>" with respect to their impact on IPV content in your program. An "internal influence" describes an effect *within* your faculty/department.** <Rank whether these influences act as facilitators or act as barriers for the delivery of IPV content in your faculty/department.>

	**Facilitator-------------------------> Barrier**				
a. Availability and access to instructors who teach IPV content	1	2	3	4	5
b. Adequate instructor preparation for teaching IPV content	1	2	3	4	5
c. The instructor's commitment to IPV content	1	2	3	4	5
d. Access to instructors with specific IPV related expertise/ research endeavours	1	2	3	4	5
e. Faculty/department commitment to IPV related education	1	2	3	4	5
f. Valid and relevant faculty/department mission or goals related to IPV	1	2	3	4	5
g. Opportunities for curriculum renewal/revision	1	2	3	4	5
h. Culture of the faculty/department	1	2	3	4	5
i. Receptiveness of education recipients to IPV content	1	2	3	4	5
j. Funding allocation for IPV education	1	2	3	4	5
k. Access to resources for delivery of IPV related content (e.g. A/V equipment)	1	2	3	4	5
l. Adequate amount of time to include IPV content in curriculum (length of program)	1	2	3	4	5
m. Other:	1	2	3	4	5
n. Other:	1	2	3	4	5

10. **Please rate the following "<u>external influences</u>" with respect to their impact on IPV content in your program. An "external influence" describes an effect *outside* of your faculty/department.** <Rank whether these influences act as facilitators or act as barriers for the delivery of IPV content in your faculty/department.>

	**Facilitator-----------------------------> Barrier**				
a. Current political climate in your institution	1	2	3	4	5
b. Current political climate more broadly	1	2	3	4	5
c. Health care restructuring	1	2	3	4	5
d. Accreditation	1	2	3	4	5
e. Licensing body approval process	1	2	3	4	5
f. External funding	1	2	3	4	5
g. Other:	1	2	3	4	5
h. Other:	1	2	3	4	5
i. Other:	1	2	3	4	5

11. **Is there anything else that we should know with respect to your IPV education?**

Contact Information

If the research team needs to contact you further, would you provide us with your contact information?

Is there an alternate contact who could discuss IPV education practices at the organization?

Institution Type

What institution type do you represent?

- ☐ University
- ☐ College
- ☐ Other: Please specify: ____

What faculty do you represent (e.g. Dentistry, Medicine, Nursing, Social Work, etc.)? Please Specify:

____

***<Thank you for completing this survey*.>**
